# A Web-Based Psychoeducational Intervention for Adolescent Depression: Design and Development of MoodHwb

**DOI:** 10.2196/mental.8894

**Published:** 2018-02-15

**Authors:** Rhys Bevan Jones, Anita Thapar, Frances Rice, Harriet Beeching, Rachel Cichosz, Becky Mars, Daniel J Smith, Sally Merry, Paul Stallard, Ian Jones, Ajay K Thapar, Sharon A Simpson

**Affiliations:** ^1^ Division of Psychological Medicine and Clinical Neurosciences Medical Research Council Centre for Neuropsychiatric Genetics and Genomics Cardiff University Cardiff, Wales United Kingdom; ^2^ National Centre for Mental Health Cardiff University Cardiff, Wales United Kingdom; ^3^ Population Health Sciences University of Bristol Bristol, England United Kingdom; ^4^ Institute of Health and Wellbeing University of Glasgow Glasgow, Scotland United Kingdom; ^5^ Faculty of Medical and Health Sciences School of Medicine University of Auckland Auckland New Zealand; ^6^ Department for Health University of Bath Bath, England United Kingdom; ^7^ Medical Research Council/Chief Scientist Office Social and Public Health Sciences Unit Institute of Health and Wellbeing University of Glasgow Glasgow, Scotland United Kingdom

**Keywords:** adolescent, depression, internet, education, preventive psychiatry, early medical intervention

## Abstract

**Background:**

Depression is common in adolescence and leads to distress and impairment in individuals, families and carers. Treatment and prevention guidelines highlight the key role of information and evidence-based psychosocial interventions not only for individuals but also for their families and carers. Engaging young people in prevention and early intervention programs is a challenge, and early treatment and prevention of adolescent depression is a major public health concern. There has been growing interest in psychoeducational interventions to provide accurate information about health issues and to enhance and develop self-management skills. However, for adolescents with, or at high risk of depression, there is a lack of engaging Web-based psychoeducation programs that have been developed with user input and in line with research guidelines and targeted at both the individual and their family or carer. There are also few studies published on the process of development of Web-based psychoeducational interventions.

**Objective:**

The aim of this study was to describe the process underlying the design and development of *MoodHwb* (*HwbHwyliau* in Welsh): a Web-based psychoeducation multimedia program for young people with, or at high risk of, depression and their families, carers, friends, and professionals.

**Methods:**

The initial prototype was informed by (1) a systematic review of psychoeducational interventions for adolescent depression; (2) findings from semistructured interviews and focus groups conducted with adolescents (with depressive symptoms or at high risk), parents or carers, and professionals working with young people; and (3) workshops and discussions with a multimedia company and experts (in clinical, research, and multimedia work). Twelve interviews were completed (four each with young people, parents or carers, and professionals) and six focus groups (three with young people, one with parents and carers, one with professionals, and one with academics).

**Results:**

Key themes from the interviews and focus groups were: aims of the program, design and content issues, and integration and context of the program. The prototype was designed to be person-centered, multiplatform, engaging, interactive, and bilingual. It included mood-monitoring and goal-setting components and was available as a Web-based program and an app for mobile technologies.

**Conclusions:**

MoodHwb is a Web-based psychoeducational intervention developed for young people with, or at high risk of, depression and their families and carers. It was developed with user input using qualitative methods as well as user-centered design and educational and psychological theory. Further research is needed to evaluate the effectiveness of the program in a randomized controlled trial. If found to be effective, it could be implemented in health, education, youth and social services, and charities, to not only help young people but also families, carers, friends, and professionals involved in their care.

## Introduction

### Prevention and Management of Adolescent Depression

Depression is common in adolescence and is associated with distress, social and educational impairments, and poor physical health. It also predicts suicide and deliberate self-harm and can mark the beginning of long-term mental health difficulties [1]. Early treatment and prevention of adolescent depression is therefore a major public health concern [2]. However, engaging young people in prevention and early intervention programs is a challenge for health and other services. This is in part because of the anxiety and stigma related to mental health issues and services, the symptoms of depression (eg, motivation and social withdrawal), and the potential difficulties in identifying depression in adolescence [1,3,4].

Guidelines for the prevention and management of depression in young people (eg, National Institute for Health and Care Excellence [5] and American Academy of Child and Adolescent Psychiatry [6]) stress the need for good information and evidence-based psychosocial interventions for the young person, family, and carer. Psychosocial interventions are likely to be important in young people for promoting resilience and preventing relapse [1,7].

### Psychoeducation

There has been growing interest in psychoeducational interventions (PIs), which deliver accurate information to individuals, families, and carers about mental health or a specific diagnosis, management and prognosis, and relapse prevention strategies [6,8-10] (Textbox 1). Although the risk factors and possible causes of adolescent depression are complex, a family history of depression, psychosocial stress, and a previous history of depression increase individual risk, and these groups could be targeted for such strategies [1].

Much of the existing literature on PIs has been in relation to individuals (mainly adults) with schizophrenia or bipolar disorder and their families [6,8,11], although there has been increasing interest in depression. Findings from a recent systematic review concluded that PIs are effective in improving the clinical course, treatment adherence, and psychosocial functioning of adults with depression [12]. We conducted a systematic review of PIs for adolescent depression [13] that showed that there were few existing programs that have been developed and evaluated using rigorous methods according to research frameworks.

### eHealth Approaches

Electronic health (eHealth) and electronic multimedia have been identified as a key area of future clinical practice and research in depression in adolescents [10,14]. There is evidence to support the use of some cognitive behavioral therapy (CBT)–based and other Web-based programs for adolescent depression [15-17]. Although it is difficult to evaluate how much psychoeducation within the programs contributed to the outcomes, Web-based PIs for depression in adults has been shown to reduce symptoms of depression and improve understanding of treatments [18].

However, to our knowledge, there is no Web-based PI that has been specifically developed for adolescent depression (or those at high risk) and developed and evaluated in line with key guidance on the development and evaluation of complex interventions [19]. This is an important gap; depression is common in young people, and its presentation and management is different to that of adults (eg, the prominence of irritability and fluctuating symptoms and emphasis on educational and psychological approaches) [1]. Studies detailing the process of development of Web-based PIs are rare. In this paper, we describe the design and development of *MoodHwb*: a Web-based psychoeducation multimedia program for young people with, or at high risk of, depression and their families and carers. The program was designed so that it could be used regularly within everyday health, social, education, youth services, and charities. This method of development provides an example and framework that could help inform the development of future Web-based programs, to ensure that they are codesigned with potential users to maximize the chances of user engagement and therefore impact.

**Psychoeducation in childhood and adolescent depression.**

The American Academy of Child and Adolescent Psychiatry (2007): “Practice parameter for the assessment and treatment of children and adolescents with depressive disorders” describe psychoeducation as “education of family members and the patient about the causes, symptoms, course, and different treatments of depression and the risks associated with these treatments as well as no treatment at all. Education should make the treatment and decision-making process transparent and should enlist parent and patient as collaborators in their own care.” (Birmaher and Brent, 2007 [6]).

## Methods

### Research Plan

The research adhered to the *preclinical or theory* and *phase I or modeling phases* of the Medical Research Council (MRC) framework for complex interventions and the *development phase* of the new guidance on developing and evaluating complex interventions [19]. The project brought together audiovisual, interactive, and Web-based media, informed by research and practice from the areas of adolescent depression (particularly prevention and management strategies), psychoeducation (and learning), e-mental health, and design.

The developmental approach was *person-centered* or *person-based*, as described in guidelines for digital health-related interventions [20-22]. This focuses on understanding and accommodating the perspectives of the users and identifying “guiding principles.” The approach embraces qualitative research, with a wide range of individuals from the target user population at every stage of the intervention development. This iterative approach helps to assess acceptability, usability, and satisfaction, as well as appreciating the context, anticipating usage and outcomes, and modifying the intervention to make it persuasive, feasible, and relevant.

**Figure 1:**
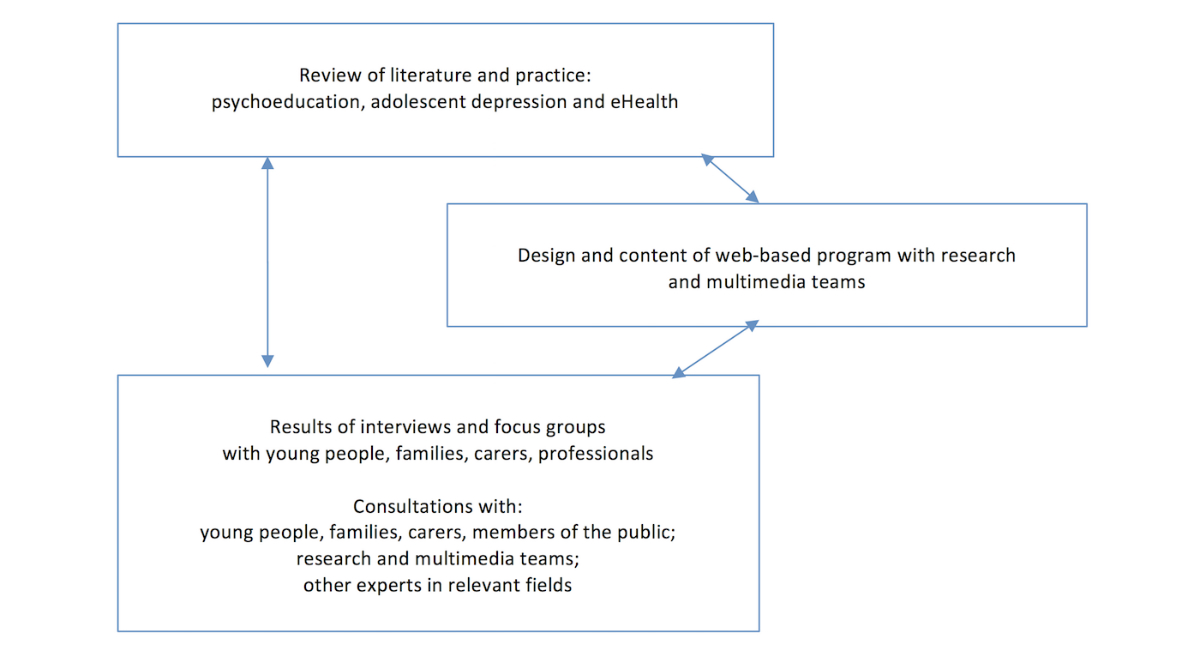
Influences on the design and content of the Web-based program.

Following a literature review of PIs in adolescent depression [13], a qualitative study was completed consisting of a series of semistructured interviews and focus groups with potential users of the program (adolescents, families, carers, and professionals working with young people). Interviews were used to generate initial detailed views and ideas from a range of users, to carry forward to the focus groups. The groups enabled a greater breadth of discussion and refinement of these ideas, to help inform the development of the program with the research team and multimedia company. The interviewing was iterative; where new themes emerged, they were incorporated into the subsequent interviews and focus groups. Figure 1 demonstrates how the design and content of the program was developed based on the literature review, interviews, focus groups, and other consultations.

### Ethical and Health Board Approval

The Dyfed-Powys, Wales, United Kingdom (UK) REC, gave a favorable opinion for the research project. Research and development (R&D) approval was granted by Cwm Tâf, Cardiff and Vale, Abertawe Bro Morgannwg, Aneurin Bevan, and Hywel Dda University Health Boards (UHBs), five of the seven UHBs in Wales.

### Recruitment for Interviews and Focus Groups

#### Inclusion and Exclusion Criteria

Young people were recruited from Child and Adolescent Mental Health Services (CAMHS) and from the Cardiff Early Prediction of Adolescent Depression (EPAD) study [23]. They were required to be at least 13 years of age and to have either current or past history of depression (recruited from CAMHS), or be at high risk of depression because of a family history (at least one parent with recurrent depression, recruited from the EPAD study).

Parents, guardians and carers of these young people were also recruited for interviews and focus groups. Professionals (from health, education, social, and youth services and charities) were approached if they worked with young people who present with mental health difficulties. Participants were not eligible if they had severe health difficulties that would make it difficult for them to contribute, or make it more likely they would become distressed, or if they were unable to understand the intervention or questions or discussions (eg, because of insufficient understanding of English).

#### Sampling Frame

Participants were recruited from south and west Wales and included areas of high deprivation, as well as more affluent areas. Within the interviews and focus groups, we aimed to have a balance of participants with regard to age, gender, primary language (Welsh or English), severity of depressive symptoms, and service involvement.

#### Recruitment Documents

**Figure 2:**
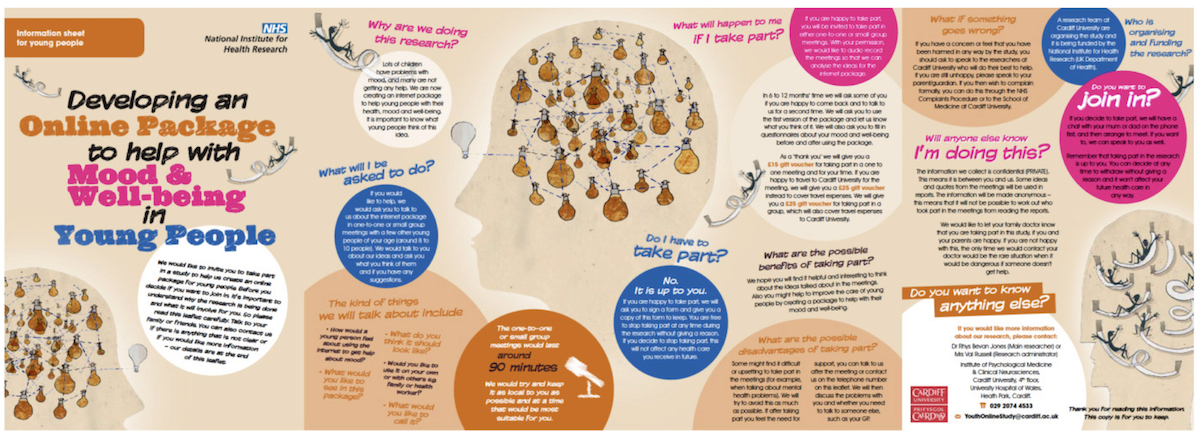
Information sheet for young people (in English).

Participants from CAMHS and the EPAD study were sent invitation letters and information sheets (Figure 2) and asked to reply if interested in participating. Young people were consulted during the design of information sheets and consent and assent forms. These were developed with reference to a readability test [24] and with the help of an experienced graphic designer so that they could be engaging for young people. The documents and the program were developed in Welsh and English in line with depression guidelines [5] and to ensure inclusivity for the population of Wales [25]. Participants were offered gift vouchers for their participation, time, and travel.

### Interviews (Discovery Phase)

A series of 12 semistructured interviews were conducted: four each with adolescents, parents or carers, and professionals. They were held either at Cardiff University or a location convenient for the participant (eg, home or school) and lasted up to 90 min. All interviews were completed by RBJ. A topic guide was used (Multimedia Appendix 1), although the interviews were informal, so that the interviewee perceived them more as a discussion. Where possible, the interviews were conducted in front of a computer screen so that initial ideas and imagery and existing resources could be discussed. All interviews were audio-recorded digitally, and a third-party company transcribed the recordings.

### Focus Groups (Codesign Phase)

Following the completion and analysis of the interviews, focus groups were conducted, which allowed new ideas to be generated in a safe forum, where participants could build on each other’s perspectives. In the earlier groups, general ideas were discussed, and in the latter groups, draft designs were shown, and participants could interact with preliminary components using tablets or laptops.

**Figure 3:**
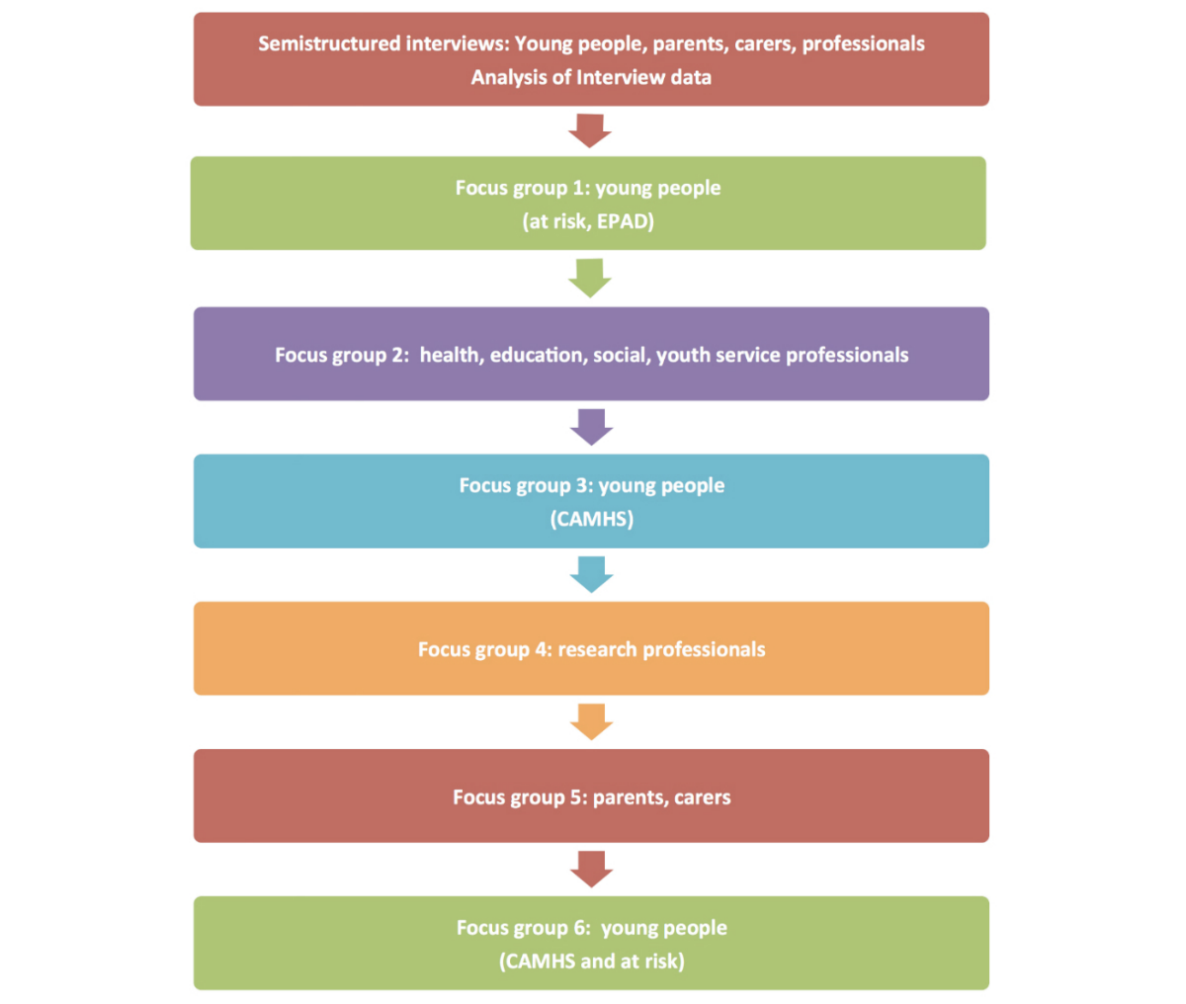
Flowchart—sequence of interviews and focus groups.

Six focus groups were held (Figure 3), three with young people, one with parents and carers, one with professionals working with young people, and one with clinical and other academics in child and adolescent psychiatry. The focus groups lasted approximately 90 to 120 min and were facilitated by RBJ, accompanied by a colleague (HB or RC). As well as engaging in a verbal discussion, participants were asked to draw or write ideas and to reflect on images and ideas presented on a screen. Each group was audio-recorded and transcribed.

The focus group discussion outline (moderator guide) evolved from the interview topic guide. Most of the questions and images discussed were projected on a screen with multimedia content (Multimedia Appendix 2). Between groups, the research team developed ideas for the presentation or content, and this shaped the multimedia slides shown to the next group. In this way, the program was developed in a staged, iterative manner.

### Other Consultations

In addition to the interviews and focus groups, there were other discussions with potential users, researchers, multimedia groups, schools, and charities from around the United Kingdom and overseas (including in national and international conferences). A workshop was held with the National Youth Assembly of Wales, Funky Dragon, a peer-led organization of young people aged 11 to 25 years elected from around Wales (n=22). There were also public engagement events where groups were consulted on their ideas for the program, such as the public engagement exhibition, How the Light Gets In [26].

RBJ also visited and consulted experts in youth health and eHealth at the Werry Institute (Auckland) and Health Promotion Agency (Wellington) in New Zealand, and the Black Dog Institute (Sydney), National Institute for Mental Health (Canberra), and Orygen, The National Centre of Excellence in Youth Mental Health (Melbourne) in Australia.

### Data Analysis

The interview and focus group transcripts were analyzed using an inductive (or “bottom up”) thematic analysis approach [27]. All transcripts were coded by RBJ and double coded independently by other authors (HB coded half of the transcripts and RC coded the other half). Initial ideas on the coding framework were discussed among the team members; the draft framework was applied to some of the data and refined as coding proceeded. Agreement on concepts and coding was then sought to ensure reliability. Where there was disagreement, the researchers reviewed the coding together. Other authors (SS, DJ, and AT) were consulted where there was uncertainty or disagreement. Codes were applied to broad themes, which were then broken down further into subcodes. Transcripts were closely examined to identify the key themes and associated subthemes. Thematic analysis was supported by the computer-assisted qualitative analysis software NVivo (QSR International) for Mac (version 10).

### Development of Prototype

Workshops were held with a multimedia company to develop the prototype alongside the latter focus groups. The specifications for the different aspects of the content and design of the program were refined according to the level of importance given to them by the participants and the potential effect on the acceptability, feasibility, and ease of use of the program. Other considerations were the technical difficulty, time required, and development costs.

**Figure 4:**
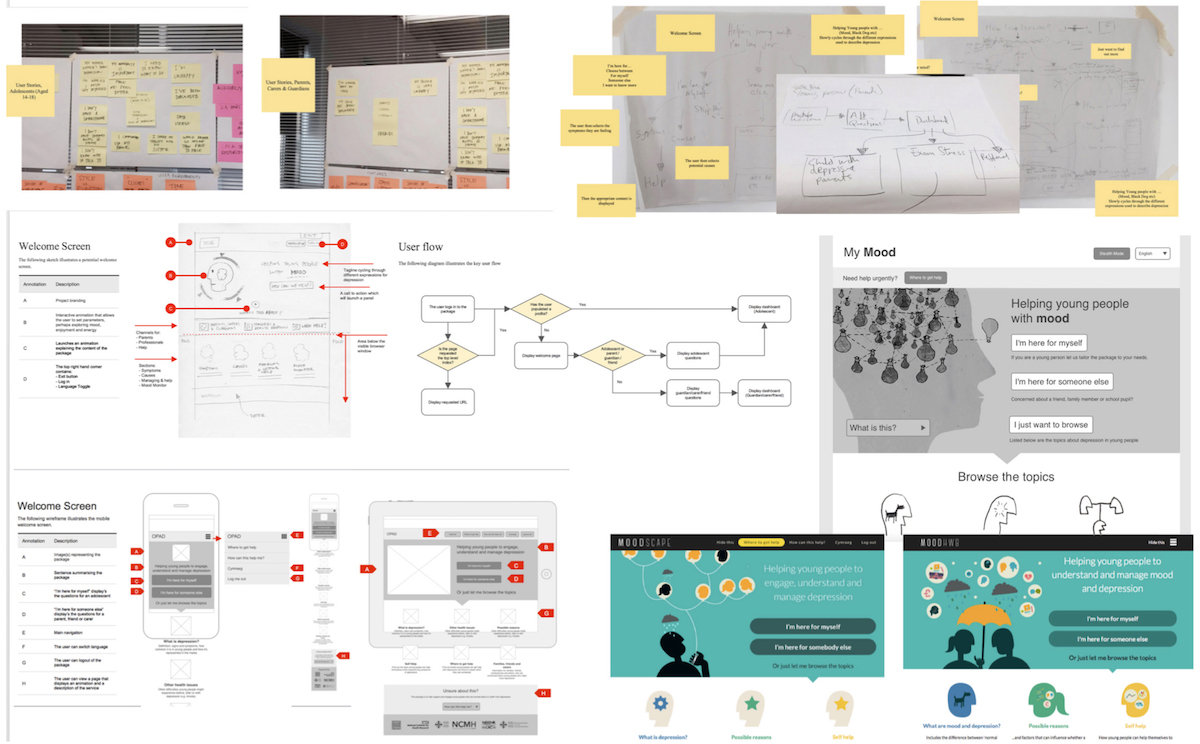
Development of welcome screen and user flow: notes or sketches (above, center left), wireframes (below left), black and white, and color designs (center and below right).

In early meetings with the multimedia company, note boards and sketches were created based on user and project requirements, and some initial designs were shown in the focus groups. Wireframes (skeletal framework or blueprints) were then constructed showing the layout and functionality of each proposed screen within the program. These wireframes further evolved into the digital prototype. The process of development from initial notes to prototype is shown in Figure 4.

RBJ wrote the script for the content and developed the initial designs and illustrations (alongside the interviews and groups), and these were reviewed by FR and discussed with other authors. During the final stages of development, components of the prototype (eg, animation scripts and storyboards) were reviewed by some of the participants who had taken part in the focus groups to ensure the material was clearly presented and age-appropriate.

## Results

### Interview and Focus Group Participants

Twelve people were interviewed in total (Table 1)—four young people aged 13 to 18 years (three females and one male), four parents (three mothers and one father), and four professionals (two child and adolescent psychiatrists, a general practitioner, and an educational psychologist).

Six focus groups were conducted (Table 2); three with young people (total N=29), one with parents and carers (N=7), and two with professionals (N=22, including one group of researchers with a special interest in child and adolescent mental health). The mean age of the participants in the young people focus groups was 16 years (range 13-19 years). The majority (69%, 20/29) were female.

### Summary of Findings to Inform the Prototype

The key themes in the interviews and later in the focus groups were (1) the need for and aims of the Web-based program, (2) design issues, (3) content issues, and (4) its integration into the young person’s life. The key themes were influenced by the topic guide and the main issues the research team wished to explore to help develop the content and design of the program. These key themes and the subthemes that emerged from interviews and groups are presented in Table 3.

#### Key Theme 1: Needs and Aims

All interview and group participants described how there was a need for this program, especially given the lack of specialist CAMHS and Web-based resources. Parents, carers and professionals also felt there was not enough time during CAMHS clinic sessions, and the waiting lists were long, as illustrated in the following quotes:

There’s a huge gap as to what else that we can offer, beyond the four life hygienes...You’ve certainly hit an area where we’ve got a big, black hole, definitely...huge, huge.

Professional 3: female: general practitioner

My daughter didn't know where to turn.

Parent 2: male: daughter under CAMHS

It’s important that people know about mental health information...There’s not much out there...it would be pretty revolutionary if it works.

Young person focus group 3

**Table 1:** Participants—semistructured interviews.

Participant group	Background	Other demographics (nationality; language)
Young person 1	18-year-old female with a history of depression (previously under CAMHS^a^)	Welsh, British; English, Welsh
Young person 2	13-year-old female with depression (under CAMHS at time of interview)	Welsh, British; English, Welsh
Young person 3	17-year-old male with depression (under CAMHS at time of interview)	Welsh, British; English, Welsh
Young person 4	16-year-old female at high risk of depression (mother has a history of recurrent depression)	Welsh, Lebanese; English, Arabic, Welsh, Spanish
Parent 1	Mother with a history of depression (14-year-old daughter is at risk)	Welsh, British; English
Parent 2	Father of a 13-year-old daughter who has a history of depression (under CAMHS)	Welsh, British; English, Welsh
Parent 3	Mother of a 13-year-old daughter who has a history of depression (under CAMHS)	Welsh, British; English, Welsh
Parent 4	Mother with a history of recurrent depression (16-year-old daughter is at risk)	Welsh, Lebanese; Arabic, English
Professional 1	Consultant child and adolescent psychiatrist (male) (advisor-National Assembly for Wales)	Welsh, British; English, Welsh
Professional 2	Consultant child and adolescent psychiatrist (male)	English, British; English
Professional 3	General practitioner (female)	Welsh, British; English
Professional 4	Educational psychologist (female)	Welsh, British; English, Welsh

^a^CAMHS: Child and Adolescent Mental Health Services.

**Table 2:** Characteristics of participants in all focus groups.

Focus group (FG)	Participants (n)	Gender (n), female:male	Age in years, mean (range)	Source of recruitment (n) EPAD^a^:CAMHS^b^	Profession
FG: young people 1 (at risk)	15	11:4	16.5 (13-19)	15:0	-
FG: young people 2 (current or history of depression)	8	5:3	15.8 (14-17)	0:8	-
FG: young people 3	6	4:2	16.8 (15-19)	2:3 (and 1 volunteer)	-
FG: parents and carers	7	7:0	-	5:2	-
FG: clinical professionals	12	8:4	-	-	2 psychiatrists, 2 psychiatric nurses, 3 educational psychologists, 2 school nurses, 1 teacher, and 2 youth workers
FG: academic professionals	10	6:4	-	-	4 psychiatrists (2 consultants and 2 trainees or fellows), 1 general practitioner, 4 research psychologists (1 senior lecturer, 2 postdoctoral, and 1 doctoral), and 1 medical student

^a^EPAD: Early Prediction of Adolescent Depression study.

^b^CAMHS: Child and Adolescent Mental Health Services.

**Table 3:** Themes from interviews and focus groups.

Key themes	Subthemes from interviews	Subthemes from focus groups
1: Needs and aims	Accessibility and target group	Increasing awareness and tackling stigma
	Increase awareness and tackle stigma	A lack of resources for young people
	Lack of resources for young people	Embrace digital technology as a medium that’s relevant for young people
	Need to engage young people	Diversity
	Embrace a medium that’s relevant for young people	Need to target parents, carers and families
	Promote self-management or autonomy	
	Accessible for a diverse range of users	
	Young people find it difficult to talk to adults	
	Help for parents, carers, and professionals working with young people	
2: Design issues	Harnessing multimedia to *engage* the user	Harnessing multimedia to engage the user
	Harnessing multimedia to *communicate* information	Multiplatform use and app
	Multiplatform approach and app	Clear structure, navigation, and distribution of information
	Clear structure and navigation	Language
	Language issues	Characters and avatars
	Characters and avatars	Imagery and metaphors
	Using metaphors to develop a relationship with the problem	Moodboards
	Color	Gaming element
	Images of heads and brains	Personalizing the space
	Gamification	Monitoring tool
	Personalizing the space	Technical aspects—security and confidentiality and use of forums or social media
	Monitoring tool	
	Forums, security, and confidentiality	
3: Content issues	General approach	General approach
	What are mood, well-being, and depression?	What are mood, well-being, and depression?
	Possible reasons for low mood and depression	Other difficulties related to low mood and depression
	Prevention and self-management	Causes, reasons and risk factors
	Where to get help	Prevention and self-management strategies
	Section for parents, carers, friends, and professionals	Where to get help
		Information for parents, carers, and professionals
4: Integration and context	Use of the program with others (eg, parent, friend, professional)	Use with parent, carer, friend, and professional
	School and education services	Use within education and health services
	Health and other services	Name, branding, and promotion
	Name, branding, and promotion	

Participants across the interviews and groups noted that using digital technologies was a valid approach to engagement, as young people use these in everyday life, although there were concerns about those without internet access:

My generation uses technologies.

Young person 4: female:at risk

It’s tapping into something they’re comfortable and familiar with.

Professional 4: female: educational psychologist

Participants in the interviews and groups stated the program could increase awareness and understanding of depression in young people, promote self-management, address the diversity in the target group, and help those who find it difficult to talk to others:

Young people struggle to benefit from sitting in a room with an adult called a psychiatrist or psychologist.

Professional 1: male: psychiatrist

It’s easier than plucking up the courage to talk to someone.

Young person focus group 2

Would rather go on internet, rather than face to face...anonymity is important.

Young person focus group 2

It was clear that there was a need to cater for parents, carers, and others concerned about a young person:

I think I would’ve liked...as we were going through it...in the beginning, I did feel that there was nobody there.

Parent 2: male: daughter under CAMHS

#### Key Theme 2: Design Issues

The overall design was discussed the most in the interviews and groups, especially by young people. This included discussions on how multimedia should *engage* users and *communicate* information:

Design is very important to teenagers…I think the visuals are everything where teenagers are concerned…Take advantage of the flexibility and complexity of multimedia.

Parent 1: female: daughter at risk

Participants suggested that the tone, language, and terminology should be at the level of the young person and discussed how much text would be appropriate. They also advised there should be clear structure and navigation:

Treating you as a mature person—not talking down.

Young person focus group 2

It should be clear what’s going on—clear where to go, what to do, clear that what you answered leads to this.

Parent and carer focus group

Most recommended tailoring or personalizing the program, for example, by using a log-in, tailoring or modifying the content according to the needs of the user, allowing the user to save or upload information, and to set goals:

It’s important that they have a say in the way they learn...You need options on how they learn that day e.g. read, do, listen etc.

Professional 1: male: psychiatrist

Everything out there’s too generalised...nothing is personal to you...one tone fits all...doesn’t feel like something you’re confident in.

Young person focus group 2

Interview participants suggested that the program should be multiplatform, and there should be a mood monitor and an app. Group participants agreed and recommended developing other interactive elements (eg, to set goals and to save links to helpful resources). The introduction of designs for the program, including elements such as illustrations, characters, metaphors, moving images, and audio, helped to guide group discussions:

If you can identify triggers—you can do something about it earlier, before letting it get to the bad points.

Young person focus group 2

What I really liked was the app, an up-to-date, modern idea.

Parent and carer focus group

It would be good to be able to give something more interactive, rather than a bunch of leaflets.

Clinical professionals focus group

Security and confidentiality were key issues in the interviews and groups, especially for young people. There were also concerns about including a forum or links with social media:

I don’t think it should be on social media—it can be so toxic.

Young person focus group 2

#### Key Theme 3: Content Issues

Participants in the interviews stated there should be different levels of information and a clear explanation of the program and its aims. There should be information on mood, the signs, symptoms and effects of depression, the difference with “normal sadness,” and related issues such as anxiety and accounts of personal experiences. Many young people and parents wished to highlight to adolescents that they are “not alone” in their experiences of depression:

When I was diagnosed and they were like you’ve got depression, I was really confused and I didn’t know what it was or what that meant for me or how that would affect my life.

Young Person 1: female: under CAMHS

Possible reasons for depression were discussed, including environmental and biological factors and also how there are sometimes no clear reasons for feeling low. Participants felt that self-management approaches should build on existing strengths and attributes:

You need to build on the positive, not magnify the downside...the depressed are quick to do this.

Professional 1: male: psychiatrist

A personalized tool kit was suggested to include short-term measures dealing with stress and healthy living:

Lots of simple solutions are usually good, these are better than complicated ones.

Professional 1: male: psychiatrist

Professionals suggested that the young person should take control and think about issues from an outsider’s perspective:

Ask them, so what are we going to do about this? Put them in charge...do you want to do something about this?

Professional 3: female: general practitioner

Participants discussed where to get help for depression, including from trusted friends and family, from school and health services, and the different possible treatments:

It’s good to talk—you don’t have to internalise everything, there are people there to approach.

Young person focus group 1

Simply—what they [the treatments] are, how they work—make it more likely that they’re used.

Young person focus group 3

They felt that there should also be information for parents or carers and others, and professionals suggested there was a need for reframing:

There’s a risk—a child wants to concentrate on positives, parent wants to say why it’s not perfect yet—child is then more depressed.

Professional 1: male: psychiatrist

Focus group participants agreed there should be levels of information, with a hierarchy of sections and subsections. The overall structure and content evolved with each group and the program prototype included sections on mood and depression, possible reasons, self-help, and where to get help. Although there were differing views, overall, it was felt there should be a separate section on “other health issues” such as anxiety, bipolar disorder, and physical illness, although there was caution:

It’s easy for the programme to become an ‘all mental health problems’ programme’.

Research professional focus group

#### Key Theme 4: Integration and Context

To help with adherence and encourage support, participants in the interviews and groups suggested it would be helpful for the young person to be able to use the program with others, including family, carers, friends, or professionals, as well as independently. The creation of a separate user pathway and section for parents, carers, friends, and professionals was supported by all focus groups—with information on signs, symptoms, and triggers; supporting the young person; and dealing with difficulties.

With regard to implementation, schools were considered an important setting for the intervention, particularly personal, social, and health education sessions. It was also felt that the program could be integrated into health and other services:

This is essential...teachers see you every day for five years!

Young person 2: female: under CAMHS

The name and promotion of the program was discussed, and the use of the term “mood” was considered more acceptable than “well-being” to young people.

### Comparison of Data From Young People, Parents, Carers and Professionals

Across the interviews and groups, young people were as interested, if not more so, in the design as they were in the content. Although parents, carers and professionals also stressed the importance of design, they were especially interested in the content. Health and research professionals stressed the underlying evidence base and guidelines for assessment and management and suggested that young people should decide on the design.

Some differences emerged in how “adults” perceived adolescent depression compared with the actual adolescent experience. Possible reasons included the adults “normalizing” of depressive symptoms and episodes, how some might not consider this to be a “real illness,” and the changing experience of young people with each generation, for example, in relation to stressors such as exam pressure, expectations, social media, cyberbullying, and parental separation.

Young people were generally positive about early versions of the program presented in the groups, and one young person stated, *It felt like it could actually do something.* Parents and carers tended to be more cautious, for example, some noted that mood monitoring might encourage a preoccupation and rumination (although monitoring can be a component of CBT for depression). To address these concerns, they suggested including alerts when the mood was low and links to sources of help. With regard to the mood boards presented in the focus groups, young people preferred the graphic and illustrative imagery, whereas parents and carers felt that the photographs would be more appealing:

They should be real, not posed or pretending to be sad, cheesy...type of thing you’d see in a school slideshow.

Young person focus group 3

Young people want real people...they can relate, connect with them...everyone wants faces.

Parent and carer focus group

Similar issues were raised in other consultations, for example, with the Funky Dragon youth group. Everyone highlighted the advantages of multimedia, given its role in the lives of young people and that the program should also target those concerned about a young person.

### Development of the Initial Prototype

#### Design and Content of Prototype

##### Structure and Functionality

**Figure 5:**
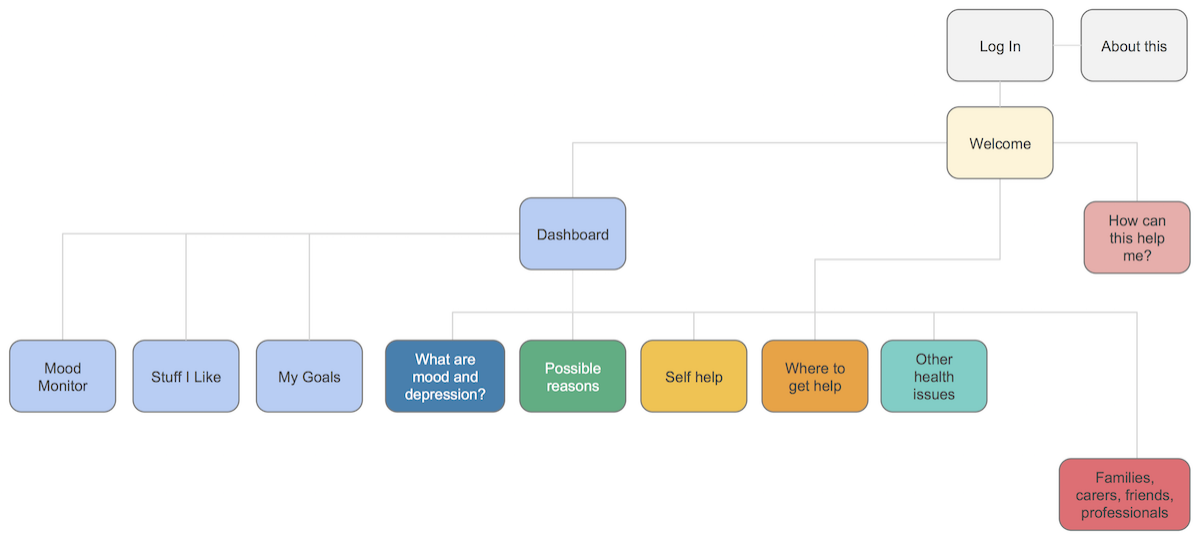
Primary level user flow diagram from the information architecture document.

Findings from the qualitative work were used to inform the development of the initial prototype. The program MoodHwb (*hwb* is the Welsh translation for *hub* and also means a *lift* or *boost*) was designed so that it could be delivered across platforms using a range of multimedia and an app. As recommended by participants, the information was developed to be clear, factual, comprehensive, age-appropriate, avoiding jargon, but not patronizing. The bilingual approach meant that the program was more inclusive and that it was designed so that it could be translated into other languages in future. Security and confidentiality were also key considerations, and this was reflected, for example, in the password-protected log-in and encryption of data. In conjunction with the multimedia company, information architecture documents were created (Figure 5) that provided an overall framework for the program.

**Figure 6:**
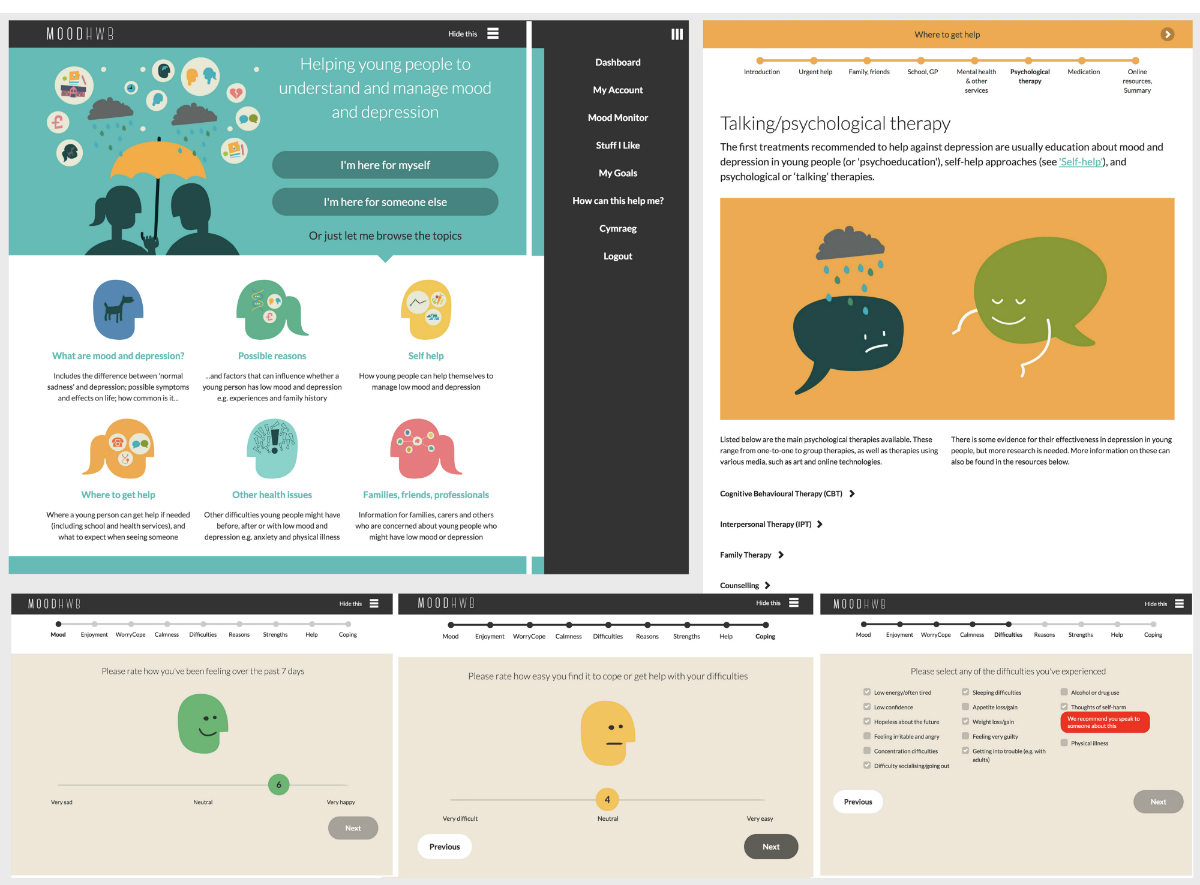
Welcome screen or menu (above left); questions when entering program or monitoring mood (below); subsection on psychological therapies (above right).

The program was designed so that it could be used independently by the young person, together with another person, or used by others to obtain information and advice. The user could personalize the content shown by choosing the relevant user pathway from the welcome screen (“I’m here for myself” or “I’m here for someone else”: Figure 6, above left).

The user would then be asked nine questions on their mood, anxiety, and other issues (Figure 6, below), and the answers helped to determine which subsections were relevant to them and were highlighted on the dashboard. If the user indicated they had thoughts of self-harm, a message would appear to advise them to seek help. These questions also enabled the user to monitor their mood or other issues over time, as the data were stored in a profile section. Along with this mood monitor, there were two other interactive elements: users could save links to helpful resources (Stuff I Like) and set goals (My Goals) by using the main site or the app.

The six main sections (“What are mood and depression?” “Possible reasons,” “Self help,” “Where to get help,” “Other health issues,” and “Families, carers, friends, professionals”) were all structured in a similar manner, so that the user could scroll through the subsections and use menus and progress bars to navigate (Figure 6, above right). This helped ensure the program looked cohesive and enabled the user to become familiar with and easily navigate it. Information was presented in levels with (1) animations and introductory or summary subsections to deliver the key messages, (2) text and illustrations in each subsection, and (3) collapsible blocks of text and links to resources for further detail. Feature blocks were used to illustrate the key information and engage the user further; these included quizzes, personal stories, questions to relate the content to the user, and links to other sites or resources. The sections were built so that the information and format could be updated and changed in response to feedback.

##### Graphics, Color, and Moving Image

**Figure 7:**
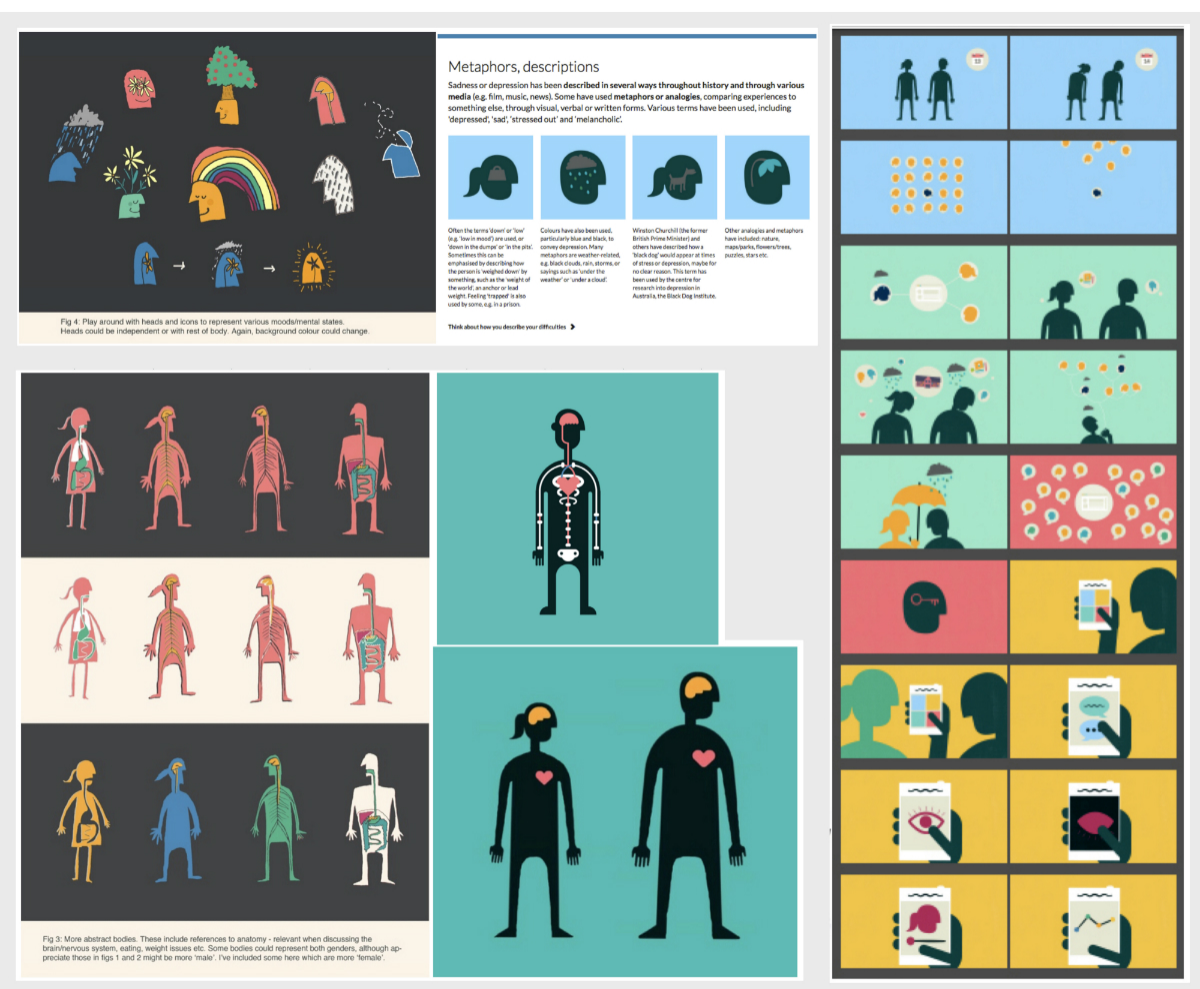
Design elements—metaphors (above left), experimental character design (below left), animation storyboards (right).

A graphic, illustrative, and colorful approach was taken for the overall design. Color was used as a tool to aid navigation—for example, blue represented the section “What are mood and depression?” and warm colors (yellow and orange) represented the help sections. The typography chosen was clear and had a distinctive character. Metaphors for depression were used to help engage the user and communicate ideas. These included “being weighed down,” “a black dog,” nature, and weather (Figure 7, above left).

The characters in the program were developed to be acceptable for a diverse range of users. A silhouette approach was deemed most appropriate, and this also allowed for graphic representations of the interior of the body, for example, when describing symptoms (Figure 7, below left). In addition, the characters needed to be clear when reducing the screen size (ie, when viewing the program on a mobile phone or tablet). An animation was developed to introduce the program and to engage the user from the start (Figure 7, right). A separate animation was also developed for each of the six main sections to communicate the key messages. The voiceovers for the animations were chosen to be warm, approachable, and bilingual, and the accompanying background music was mellow but uplifting.

### Theories and Approaches Underpinning the Program

Many theories and approaches were suggested in the interviews and groups with professionals. Some theories and approaches were also mapped onto the suggestions for the design and content made by young people, parents and carers, with reference to relevant literature (Multimedia Appendix 3).

#### Design and Educational Theories and Approaches

A key issue relating to the program was that it should be person-centered and follow guidelines for the development of digital health-related interventions [20-22]. The framework by Mohr et al [21] describes considerations for the theoretical approaches (aims [“why”] and conceptual or behavior change strategies [“how”]), as well as the instantiation and technical approaches (elements [“why”], characteristics [“how”], and workflow [“when”]). Another approach followed was the Persuasive System Design [22] that identifies four general design features: (1) primary task support, including tailoring or personalization and self-monitoring; (2) dialogue support, including positive reinforcement and suggestions; (3) credibility, by conveying trustworthiness and expertise, in the general tone and references to evidence and guidelines, supporting institutions, and user input in the development process; and (4) social support, by encouraging use and discussions with a trusted person and providing links to other sources of help.

The program was personalized where possible, so that engagement was a two-way process, whereby the participants could take ownership of the program rather than be a passive recipient of information [28,29]. Diversity and learning preferences are key considerations in education, and the program was developed to be accessible and inclusive. A range of media helped to engage and adapt to the user’s learning style or preference, and key themes and messages were repeated in various ways [30]. This linked in with the VARK approach, which states that individuals prefer to learn through different sensory modalities: visual, auditory, read or write, and kinaesthetic.

Other theories and approaches that informed the development of the program included a blended learning approach, Bloom’s taxonomy, Kolb’s experiential learning theory, the constructivist approach, and approaches to help with concentration, such as segmenting sections and interactive elements [31-34].

#### Psychological Theories and Approaches

Information on talking or psychological therapies for depression was presented in the help sections. Elements of CBT were suggested by professionals, possibly because many practiced this and because this approach is recommended in guidelines for treating depression [5]. CBT theory was referred to in the program when discussing possible symptoms, effects, reasons, negative thoughts, and metaphors related to mood, depression, and other issues. The goal-setting component and self-help subsections used elements of behavioral activation theory [35].

Aspects of positive psychology were incorporated in the general tone of the program, particularly where the user was encouraged to think of their “strengths and positives,” and how these can help them to overcome difficulties. Interpersonal and family systems theories were referenced in help sections, encouraging the user to consider their relationship or roles with friends and family and those advising others to support the young person.

The development and content of the intervention was influenced by several behavior change theories including information, motivation, behavior theory, self-regulation theory, self-determination theory, and social cognitive theory [36-38].

### Logic Model

A logic model (Multimedia Appendix 4) was developed based on the initial qualitative work, a literature review, and relevant theory. There were a range of possible areas of change that could be targeted by the program and a range of possible outcomes. The model described the inputs in terms of intervention components, the mediators of change or mechanisms, and the intermediate and long-term outcomes, as well as contextual factors that might influence the effectiveness (or otherwise) of the intervention. The model gave a reference point while developing the various elements of the program and not only informed the content of the program (especially the information in the six main sections) but also the way in which it was designed (eg, the different user pathways). The logic model was critical in the development of the program as it demonstrated mechanisms by which the intervention would be expected to work to support young people and gave a framework which could be tested with mixed methods approaches in the evaluation phase.

## Discussion

### Summary, Integration, and Context

#### The Web-Based Program MoodHwb

This manuscript describes the collaborative design and development of a Web-based psychoeducation program for adolescent depression (MoodHwb, or HwbHwyliau in Welsh). This program could help young people with (or at risk of) depression, as well as their families, carers, friends, and professionals. It has the potential to be integrated into a range of services including health, education, social, and youth services and charities.

MoodHwb meets the need outlined in the guidelines for depression in young people [5,6] for good information and evidence-based psychosocial interventions for the young person and family or carer. The review conducted as part of this work on PIs for adolescent depression [13] concluded that there were few existing programs, particularly Web-based, that have been developed and evaluated using rigorous methods. This program will help to fill this important gap.

The program also fits with the UK government’s push to provide access to Web-based therapies to young people through information and communication technology [39-42]. Over 90% of young people aged 16 to 24 years have internet access in their homes [43], and the use of technological devices (such as computers and smartphones) is similar in individuals with mental health disorders compared with the general population [44]. eHealth approaches offer a valuable opportunity to provide a novel intervention widely and at low cost compared with face-to-face therapies. Web-based interventions also address issues of accessibility, waiting lists, and treatment flexibility.

The findings from the development work suggested the program might be particularly helpful in the early stages of depression when a young person starts to experience difficulties. This could be when they first present to professionals in education, health, or other sectors. MoodHwb could be integrated into each of these services at the lower levels of the stepped care approach for depression in young people [5]. It could also complement (and be an adjunct to) other approaches, for example, in the management of more severe or chronic difficulties.

MoodHwb fits with the guided self-help approach as it has been designed so that it can be used either independently or with another person. PIs could be delivered by a range of professionals, and Colom [9], a pioneer in psychoeducation for mood disorders, states that facilitators need to be “an expert on the disorder not the technique,” avoiding the “complex training” and associated funding required, for example, for CBT. Web-based PIs could therefore help in areas where there is a lack of skilled alternative approaches, including in low and middle-income countries [45].

#### The Development Process

Historically, pressure to identify effective interventions in public health has led to many interventions being tested for effectiveness in randomized controlled trials (RCTs), with little rigorous development work being completed [46]. This has led to large amounts of resources being invested in interventions that are unlikely to be effective. However, frameworks for the development and evaluation of complex interventions [19] stress the importance of the development phase, which is at least as important as the effectiveness evaluation stage. Proper development involving users, relevant theory, and research evidence is more likely to produce an intervention that is acceptable and feasible to deliver and also potentially effective. This process also facilitates identification of potential problems that may be preventable at a later stage (eg, in a large trial).

In the world of digital health, this development phase is also crucial, and user-centered design is a common approach used [20-22]. As more digital health apps and interventions are being developed, one issue that is important to consider is whether these interventions should be tested in RCTs or whether other evaluation methods should be used. Given the pace of development of digital health, evaluation methods are needed that are more agile and more user-centered than the standard RCT methodology, which may take too long to produce the results needed. However, when feasible, RCTs represent the most internally valid means of establishing the effectiveness of complex public health interventions [47]. In the MRC guidance, emphasis is placed on conducting randomized trials of such interventions [19]. The staged, user-centered approach to development taken here is recommended regardless of whether the effectiveness is evaluated in an RCT or using other methods of evaluation.

There is a lack of literature not only on the overall development process of Web-based (PI) programs but also specific elements such as the approach to graphic design or illustration in the context of youth mental health. This paper gives a detailed account of the process of development and a practical example of how to follow existing guidelines and could help inform other studies and programs in this field. The paper stresses the importance of involving all potential users (young people and their families, carers and professionals) in the development of all aspects of the program—not only the content but also the design and technical elements and why this is crucial especially when attempting to engage young people with mental health issues. The underlying theories associated with all these aspects were considered (not only the psychological theories), and this influenced the development of the logic model. The researchers were also involved in all stages and in regular discussion with the multimedia company and other agencies.

### Comparison With Other Web-Based Programs

There are few studies that describe the process of development of Web-based PIs to give context to this study. However, there was some overlap in the content of MoodHwb and existing Web-based or computerized PI programs. For example, the program The Journey by Stasiak et al [48] also covered depression, “mental health hygiene,” and stress reduction. Furthermore, Demaso et al [49] found that personal stories were well-received in their feasibility trial of the Depression Experience Journal. This Web-based program, and the one described by Stjernswärd and Hansson [50], stressed the importance of supporting the families and carers of those with depression.

The graphic illustrative and animated approach favored by the participants in this study is different to the approach taken by many youth mental health sites (eg, YoungMinds, Childline (United Kingdom), Headspace (Australia), and The Lowdown (New Zealand) [51-54]) that are based largely on photographs of young people—although some have used illustrations and characters. When showing examples of other sites to the young person groups, they noted that the most attractive resources were those that were clear, colorful, graphic, and uplifting in their design and content, such as Headspace (United States); a mindfulness resource that also uses characters, imagery, and visual metaphors [55].

Web-based CBT and other therapeutic programs for adolescent depression incorporate a range of elements, from mainly text-based to image-based designs [16,17]. Some of the themes discussed by participants for this project (eg, characters, avatars, and gamification) also applies to the computer game CBT program, SPARX [15]. The researchers behind this intervention also consulted young people in its development.

### Strengths and Limitations

#### Strengths

One of the main strengths of the project relates to the rigorous methodology, which followed the MRC guidance for complex interventions and the consultative and person-based approach [20]. Interviews and focus groups progressed in an iterative fashion so that participants could guide the development. The interview and group transcripts were all analyzed and double coded to ensure reliability of coding.

Another strength was the diverse range of participants, including young people from mental health services (primary and secondary care) and volunteers, many of their parents and carers, and a mix of professionals. Young people at high risk were also recruited from a previous study sample. There were a range of recruitment centers in urban and rural areas, including in areas with several ethnicities and areas of deprivation. Participants were also recruited from communities where Welsh was spoken widely.

The results from the review, interviews, and focus groups were complemented by other investigations, including advice from experts, visits to centers of excellence in youth mental health and eHealth in the United Kingdom and overseas, and workshops with multimedia professionals. The development was further strengthened by using user-centered design, educational and psychological approaches, and a logic model, as well as basing the content and design on current research evidence and guidelines. All these approaches helped to create a potentially accessible, engaging, and informative program that the user could personalize, which fits with the increasing interest in precision (personalized) medicine [56]. The detailed account of the development process could inform the development of other programs in the future.

#### Limitations

The research or multimedia teams were involved throughout the project, and it is possible that their personal views could have influenced the process of development. However, a range of different groups was consulted, which would help minimize bias. Certain decisions regarding development were made before the interviews and groups (eg, that this would be a Web-based resource targeting adolescent depression), and the key themes that emerged may have been influenced by the topic or discussion guide. However, it was necessary to have some focus to the discussions, and this was not intended as an open scoping exercise. All prior decisions were based on a literature review and expert clinical opinion.

As with all methods of data collection, there are limitations to the interview and focus group approach. Although efforts were made to recruit a diverse sample with different backgrounds and experiences of depression, the overall number of participants was small and may not be representative of the population of adolescents who are experiencing depression or who are at risk. In addition, it is possible there was self-selection, in that participants who volunteered were more likely to have an interest in the project. Although the number of initial interviewees (n=12) was small, many ideas and themes were repeated as the interviews progressed, indicating that saturation might have been reached. The participants were purposively chosen at this early stage of the project to offer a range of viewpoints or ideas to guide later focus groups.

Focus groups were balanced as far as possible with regard to age, gender, and experience of mental health difficulties. However, the number of those who attended the focus groups varied across the study. Recruitment for young person groups 2 and 3, where the majority had experience of mental health difficulties, was difficult. Some participants cancelled at short notice or did not attend, although this is not unusual for focus groups [57,58]. In addition, three fathers had stated they would attend the groups but did not, leading to an all-female parent and carer group. This is consistent with other depression studies involving families, where most participants were mothers [23].

### Conclusions

A Web-based psychoeducation program was coproduced to help adolescents with or at high risk of depression and their families, carers, friends, and professionals. The program MoodHwb was developed following rigorous methods. This included a review of the literature, interviews and groups with potential users at all stages, and consultations with experts and with a multimedia company.

An early evaluation is planned to assess whether the program (and its assessment) is potentially acceptable, feasible, clear, and easy to use. Young people (and their parents or carers) will be recruited from CAMHS, the EPAD sample, primary mental health services, and school counselors and nurses. Participants will complete questionnaires before and after using MoodHwb, a subsample will be interviewed to collect feedback on the package and the study process, and there will be a focus group for professionals. Web usage will also be analyzed.

The results will inform the future development of the program. A feasibility trial would then be completed in line with the MRC guidance [19]. A key focus of future work would be to develop and test the logic model to examine the mechanism of action and active components of the intervention. The program would also be developed further in response to the feedback from each evaluation phase, and this is particularly important when considering the advances in technology and health research. If MoodHwb is proved to be effective, it could be rolled out in health, education, youth, and social services and charities.

## Appendix

Multimedia Appendix 1 Topic guide for the semistructured interviews.

Multimedia Appendix 2 Questions and selection of slides shown during a focus group.

Multimedia Appendix 3 How interview and group findings influenced the development of the program.

Multimedia Appendix 4 Logic model.

## Abbreviations

CAMHS: Child and Adolescent Mental Health Services
CBT: cognitive behavioral therapy
eHealth: electronic health
EPAD: Early Prediction of Adolescent Depression
MRC: Medical Research Council
PI: psychoeducational intervention
RCT: randomized controlled trial
