# Supplementary material for: A Web-Based Psychoeducational Intervention for Adolescent Depression: Design and Development of MoodHwb
Source: JMIR Ment Health. 2018 Feb 15;5(1):e13. doi: 10.2196/mental.8894 (PMC5832901; doi:10.2196/mental.8894)
Supplement: Multimedia Appendix 1 [file mental_v5i1e13_app1.pdf]

Topic Guide – for the semistructured interviews

(Some topics were more relevant for certain groups eg, adolescents, parents, carers, professionals)

Opening questions:

How would you use the Internet to get help?

What do you look for when looking for information or help?

Any general thoughts about the development of a web-based psychoeducation program?

How would a young person feel about using the Internet to get help about mood?

What would you like to see in this program?

a. Various sections eg, diagnosis, investigations, management

b. How would you differentiate this from psychotherapies such as CBT?

What do you think it should look like?

a. What would be the various formats? Eg, visual, audio, animation, interactivity?

b. What would be the X-factor or essential ingredient for it to work?

How would you personalise it?

Should it be used alone or with others eg, family or health worker?

How would you monitor your mood or scores?

How would you ensure it would integrate into services?

a. Health b. Social c. Education d. Youth

What do you think might be the difficulties?:

a. in its development, b. in it being used eg, side effects

What would you like to call it?

Any thoughts about the examples shown, and any elements which could help develop the program?

eg, Beating Bipolar, YoungMinds, Headspace, other graphic work

Any other comments/questions? Thank you for participating
