# Supplementary material for: A Web-Based Psychoeducational Intervention for Adolescent Depression: Design and Development of MoodHwb
Source: JMIR Ment Health. 2018 Feb 15;5(1):e13. doi: 10.2196/mental.8894 (PMC5832901; doi:10.2196/mental.8894)
Supplement: Multimedia Appendix 2 [file mental_v5i1e13_app2.pdf]

Questions and selection of slides shown during a focus group

Aim of project: To develop a web-based program for mood and well-being in young people (aged 14-18 years) and their families and carers

Aim of group meeting: To discuss ideas for the content and design of the program and related issues

Why should I help?
- Program will benefit young people and families
- Opportunity to develop a program rolled out across the UK
- £25 gift voucher for taking part

Ground rules
Everyone to contribute (some more familiar with study than others)
One person to talk at a time
Respect each other's comments
No wrong answers
Not asking about personal experiences
Discussion not to go outside of the group
You may leave at any time (please let us know)
Any questions?

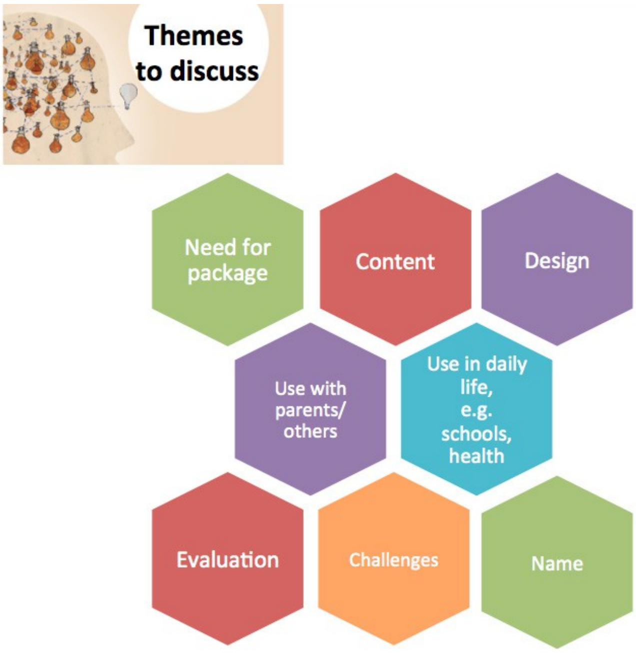

Initial questions
How many here access the Internet regularly?
How many use the Internet to look up information on health and mental health?
How many would use an web-based program for mood and well-being?
Briefly explain:
why you are interested in this study
and/or whether this program is needed and why (or any general comment about the study)

Structure and content of program
The content and structure was discussed, with a general initial question on what information should be included in the program – then each of the modules or circles below would appear in sequence.

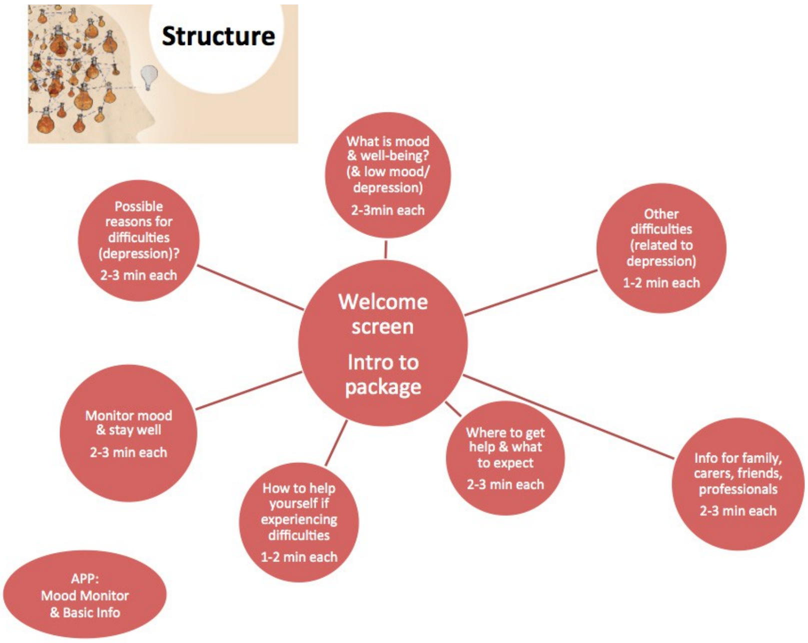

Design issues
The specific design issues of the program were then discussed in sequence.

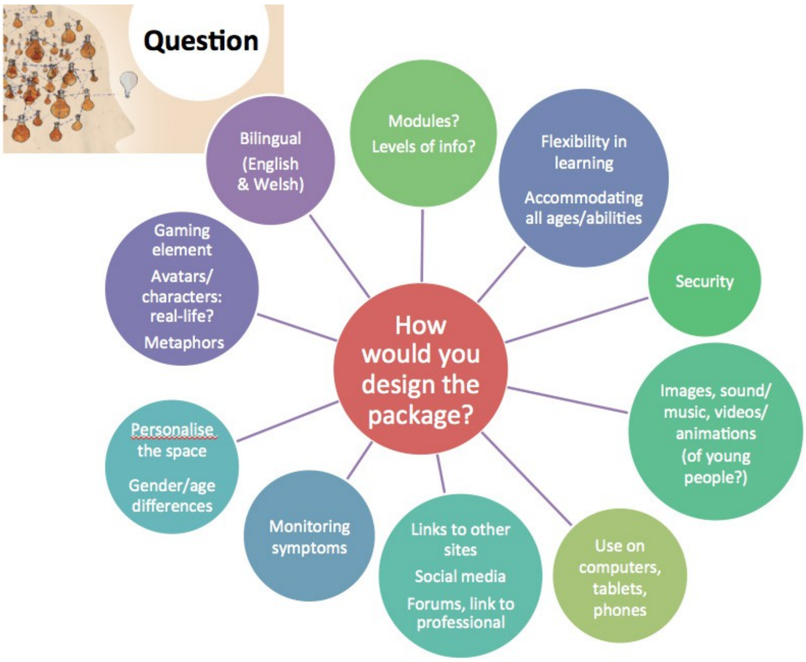

Other topics
Finally, there were additional questions regarding the program's use with others, what would make the person use the program, how would it be evaluated, the challenges and side-effects and the potential name for the program.

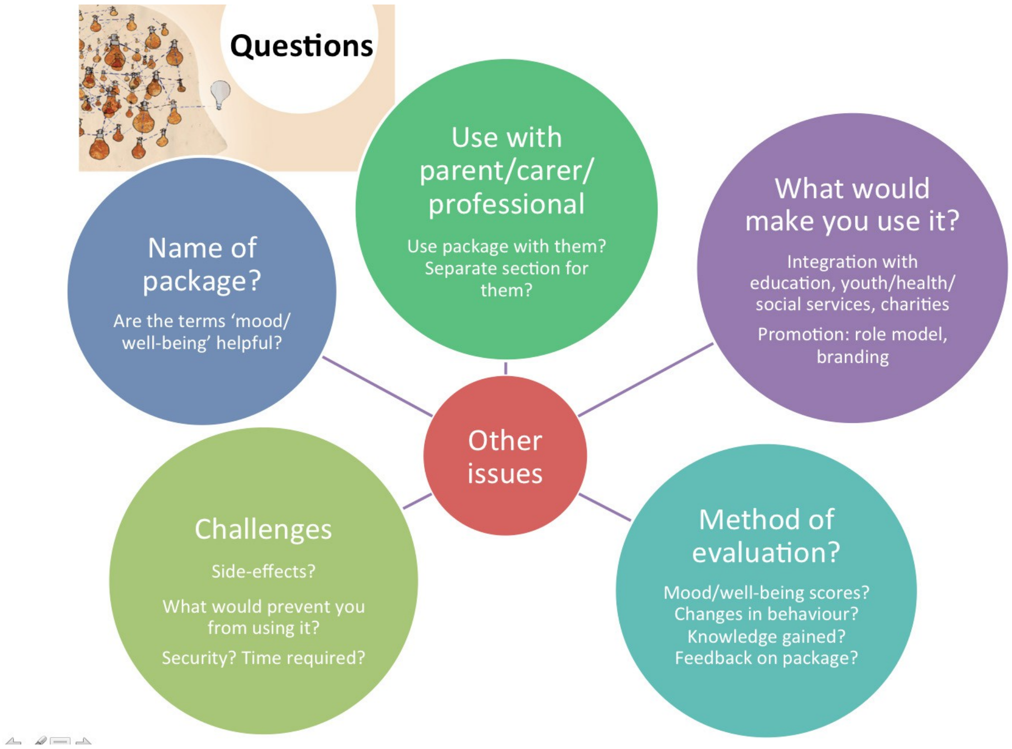

Examples of educational programs
Examples were then shown of educational healthcare programs and websites to help inform the current project, with certain components of the programs shown, and participants asked to respond to aspects of these. These included Beating Bipolar (beatingbipolar.org), Spring (myptsd.co.uk), YoungMinds (youngminds.org.uk), Headspace (headspace.org.au), Mindfull (mindfull.org), and Headspace (headspace.com).

The **final slides** allowed participants to raise any other issues and ask questions. Participants were thanked, and directed to resources on depression and other difficulties. They were informed that they could write any comments (or draw any images) if they did not feel able to do so during the focus group itself, and leave them in a box in the room. They were also informed that the facilitators would be available after the meeting to discuss any issues, particularly if they felt distressed or concerned about any issues arising from the meeting.
