# Supplementary material for: A Web-Based Psychoeducational Intervention for Adolescent Depression: Design and Development of MoodHwb
Source: JMIR Ment Health. 2018 Feb 15;5(1):e13. doi: 10.2196/mental.8894 (PMC5832901; doi:10.2196/mental.8894)
Supplement: Multimedia Appendix 3 [file mental_v5i1e13_app3.pdf]

Table: How the interview and group findings influenced the development of the program

Findings from interviews and groups	Examples of how themes were addressed in the web-based program	Possible underlying psychological and behaviour change theory and approach
**Key theme 1: Needs and aims**		
Accessible for a diverse range of users	Introduce 3 broad user pathways: i) young person, ii) parent, carer, other, iii) browse option;<br>Develop design elements (eg, character design) which are not specific eg, to gender, age, culture;<br>Implement different levels of information and personal stories from a range of perspectives	
Increase awareness, knowledge, understanding;<br>Reduce stigma	Communicate information on mood and depression at an accessible, youth-friendly level;<br>Explain that low mood and depression is common in young people, and it can affect everyone	IMBT
Embrace how young people use digital technologies	Develop the program so that it can be used on a range of platforms, especially portable devices;<br>Develop an accompanying app	SDT, SRT
Promote self-management or autonomy	Give opportunities for the user to relate the content to their experiences eg, through interactive features;<br>Develop a Self help section	SDT, SRT
Young people find it difficult to talk to adults	Develop the program so that it can be used alone or with someone the user trusts;<br>Provide a diverse list of resources for the user and highlight a variety of different sources of help	IMBT
Help for parents, carers and professionals working with young people	Develop user pathways specifically designed for families, carers, friends and professionals;<br>Develop a separate section for them as well	SCT, IMBT, Resilience theory, Family systems theory

**Key theme 2: Design issues**		
Harnessing multimedia to:<br>i) *engage* the user, ii) *communicate* information	Use a range of imagery and multimedia formats such as animation and audio	
Clear structure and navigation, multiplatform use	Develop clear user pathways from the welcome screen, use drop-down and side menus;<br>Keep the structure of the modules consistent;<br>Use color to easily distinguish between sections and aid navigation	
Language	Create a bilingual program (English and Welsh);<br>Ensure the general tone and wording are age-appropriate;<br>Avoid use of jargon and ‘unhelpful’ words and phrases	
Characters	Develop engaging characters with some ambiguity, so that they are not specific to gender, age, culture;<br>Ensure that facial expressions are used sparingly in the illustrations and animations	
Imagery, metaphors, moodboards, color	Use images and visual metaphors to help illustrate aspects of mood and depression throughout;<br>Take a graphic illustrative approach throughout, rather than photography and live-action video;<br>Ensure the colors are bright and uplifting, and appropriate for the general design, color contrast etc.	CB theory
Gamification	Introduce a quiz at the start and the end of each section	
Personalising the space,<br>monitoring tool, interactive elements	Ask the user to answer questions at the start of the program (and throughout) to:<br>i) help monitor mood and other issues, ii) highlight relevant subsections within the program;<br>Encourage self-monitoring, in a helpful manner;<br>Develop a ‘My profile’ component, including answers to questions, allow user to select profile picture;<br>Develop a ‘Stuff I like’ component and app;	SRT, SDT,<br>CB theory, Behavioural activation

	Develop a ‘My goals’ component and app	
Security and confidentiality<br>(Forums, social media)	Ensure the user’s account is password-protected;<br>Encrypt account data on servers;<br>Do not link to forums or social media, or allow users to share information with one another	
**Key theme 3: Content issues**		
General approach<br>*Levels of information*	Communicate general information to young people and others regarding aspects of mood and depression;<br>Introduce levels of information to the sections:<br>i) animations (and intro and summary subsections) for key messages,<br>ii) subsections to include text and image,<br>iii) further details via collapsible blocks, feature blocks,<br>iv) links to further resources	IMBT
Positive and validating approach	Emphasis on strengths and positives throughout, not only possible difficulties;<br>Subsections in ‘Self help’ section eg, ‘Believe in yourself’	SDT, SCT, Resilience theory,<br>Positive psychology
Mood, depression, its symptoms and effects	Communicate information on: what are mood and depression in young people;<br>Take a bio-psycho-social and cognitive behavioural approach–identify links between thoughts, feelings, actions;<br>Include personal experiences – diversity in case studies, recovery oriented	IMBT,<br>CB theory
Possible reasons for depression	Communicate information on: possible reasons and triggers, how to appreciate strengths, focus on modifiable factors;<br>Include a diverse range of possible reasons for depression, taking a bio-psycho-social approach	IMBT,<br>CB theory
Prevention and self-management	Communicate information on: range of self help strategies;<br>For self-efficacy – subsection on ‘Believe in yourself’;<br>Include some information on sleep, exercise, diet, drugs and alcohol	SDT, SCT, IMBT,<br>CB theory, Resilience theory,<br>IP theory, Family systems theory

Where to get help	Communicate general information on: range of sources of help (eg, in an emergency);<br>Include information on what to expect from services;<br>Links to helpful resources;<br>Information on psychological treatments and medication	SDT, IMBT,<br>IP theory,<br>Family systems theory
Other health issues	Build a separate section for other health issues eg, anxiety, self-harm;<br>Communicate general information for each and provide links to further resources	SDT, SRT, IMBT,<br>CB theory
Parents, carers, friends, professionals	Develop a section specifically for parents, carers and friends;<br>Link this section to the adolescent sections for further information;<br>Develop personal stories from perspective of others	SCT, IMBT, Resilience theory,<br>IP theory,<br>Family systems theory
**Key theme 4: Integration and context**		
Use of the program with others	Develop the program so that it could be used with someone else	SCT, IMBT
School and education services	Develop the program so that it could be used independently by pupils, or with school counsellors or in lessons	SCT, IMBT
Health and other services	Develop the program so that it could be used with health professionals	SCT, IMBT
Name, branding	Use a name and approach that resonates with young people, with no negative or stigmatising associations	

Abbreviations: Cognitive behavioural theory (CB theory); Interpersonal theory (IP theory); Information, motivation, behaviour theory (IMBT); Self regulation theory (SRT); Self determination theory (SDT); Social cognitive theory (SCT)
