# Supplementary figures and images for: A Web-Based Psychoeducational Intervention for Adolescent Depression: Design and Development of MoodHwb
Source: JMIR Ment Health. 2018 Feb 15;5(1):e13. doi: 10.2196/mental.8894 (PMC5832901; doi:10.2196/mental.8894)

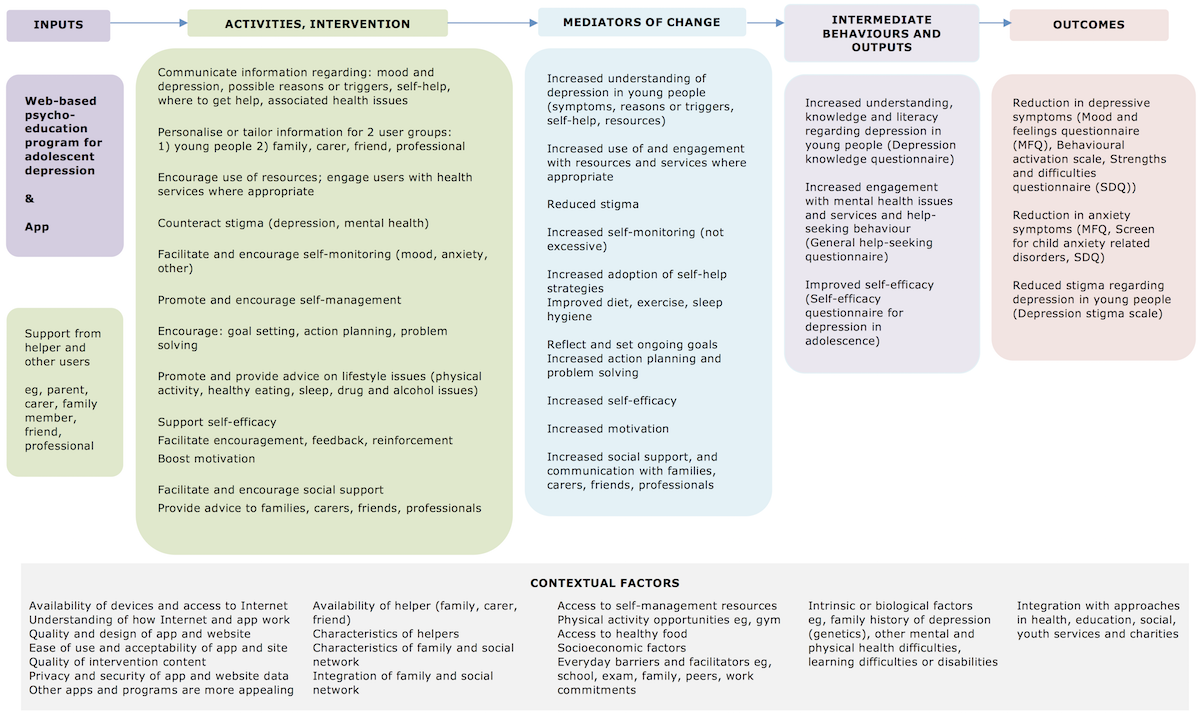

Supplement: Multimedia Appendix 4 [file mental_v5i1e13_app4.png]
